# Effects of occupational exposure to dust on chest radiograph, pulmonary function, blood pressure and electrocardiogram among coal miners in an eastern province, China

**DOI:** 10.1186/s12889-019-7568-5

**Published:** 2019-09-05

**Authors:** Qiuyun Wu, Lei Han, Ming Xu, Hengdong Zhang, Bangmei Ding, Baoli Zhu

**Affiliations:** 1 School of Public Health, Xuzhou Medical University, Xuzhou, 221004 China; 2 Institute of Occupational Disease Prevention, Jiangsu Provincial Center for Disease Control and Prevention, Nanjing, 210009 China; 3 Center for Global Health, School of Public Health, Nanjing Medical University, Nanjing, 211166 China

**Keywords:** Occupational health, Miner, Dust, Physical examination

## Abstract

**Background:**

Coal dust is one of the most serious risk factor that leads to respiratory diseases and cardiovascular diseases in miners. This study aimed to observe the effects of occupational dust exposure on chest radiograph, pulmonary function (PF), blood pressure (BP) and electrocardiogram (ECG) indexes in coal miners and explore the related risk factors.

**Methods:**

In the Chinese Occupational Disease Monitoring and Occupational Health Risk Assessment Program, a total of 11,061 subjects in 2015 and 12,597 subjects in 2016 were recruited in this study. The chest radiograph, PF, BP and ECG of coal miners were surveyed using radiograph machine, spirometer, sphygmomanometer and electrocardiograph, respectively.

**Results:**

The prevalence of aberrant BP was the highest in coal miners, followed by abnormal ECG, PF and radiograph. Significant differences in abnormal BP, ECG, PF and radiograph of coal miners were closely associated with age, years of dust exposure, smoking, drinking, working types and size of mines. A total of 80 persons diagnosed with coal workers’ pneumoconiosis (CWP) in 2015–2016, which occupied 0.34% of the coal miners.

**Conclusion:**

Abnormal BP, ECG, PF and radiograph of coal miners are highlighted health problems in China and require serious attention. Feasible health promotion and protective facilities should be adopted to guarantee coal miners’ health.

**Electronic supplementary material:**

The online version of this article (10.1186/s12889-019-7568-5) contains supplementary material, which is available to authorized users.

## Background

China has a high record of coal mine production and consumption, and various hazards significantly threaten coal miners’ health, such as dust, noise, vibration and high intensity work [1]. Among these, the most serious risk factor is coal dust, which contains crystalline forms of silica (SiO_2_). Exposure to coal dust for a long time can lead to the formation of large nodes and diffuse fibrosis [2, 3], which significantly decrease lung function and consequently cause coal workers’ pneumoconiosis (CWP) [4, 5]. Owing to its progression and irreversibility, no specific medicine and effective treatment are suitable for CWP. So the patients always lose labor ability, reduce life quality and shorten life expectancy [6, 7].

The risk of CWP was positively related with the increased exposure to coal dust, so the prevention of dust exposure is a crucial work. Technical methods to decrease dust concentrations in the workplaces are the most effective measures, such as wet working and cleaning dust [8]. The regular measurements to monitor the total and respirable dust in the workplaces are still the essential supervision methods. In addition, the routine occupational health examinations are the crucial measures to monitor and protect coal workers’ health.

The Chinese government has paid close attention to coal miners’ health in order to prevent CWP. Since 2015, the Chinese Center for Disease Control and Prevention has administered the Occupational Disease Monitoring and Occupational Health Risk Assessment Program to study the characteristics and prevalence of silicosis and CWP, which mainly collected information about occupational health examination and diagnosis of silicosis and CWP in coal miners. Furthermore, the law of Occupational Disease Prevention and Treatment in China establishes a compulsory provision on the occupational health examinations of coal miners. It means that the legislation provides a routine chest radiograph examination to ensure miners’ lung health [9]. Because the lung function impairment is the main feature related to dust exposure, the occupational examinations have added the spirometry testing together with the chest radiograph in China. In addition to respiratory diseases, abnormal blood pressure and cardiac function are also identified as the main health problems of dust-exposure workers. One previous study found that the blood pressures increased significantly over the 5-year follow-up cohort of copper-gold mining workers [10]. Furthermore, the hypertension and obesity were prevalent in US coal miners [11]. All of these suggested a further need for cardiovascular health management in coal miners, but there is no data about the prevalence of abnormal blood pressure and electrocardiogram in coal miners in China.

To our knowledge, few studies have systematically analyzed the results of occupational examinations in Chinese coal miners so far, and reference data on abnormal health outcomes and related influencing factors are rather limited. To further improve health management, we mapped results from occupational examinations of coal miners to display the prevalence of abnormal chest radiograph, pulmonary function, blood pressure and electrocardiogram in 2015–2016 and explore the related risk factors. This study will provide evidence to facilitate the development of coal miners’ health management regarding exposure characteristics, and provide direction for future researches to evaluate the health hazards of coal miners.

## Methods

### Data sources and study participants

The health surveillance data were collected from mines in Jiangsu, China between January 1, 2015 and December 31, 2016. A total of 11,061 participants in 2015 and 12,597 participants in 2016 were recruited in this study. The major contents included age, sex, smoking, drinking, years of dust exposure, working types and size of mines (see Additional file 1). The working type of each participant was categorized according to the mining sector in which they worked, including tunneling, coal extraction, pulverized coal, underground auxiliary and ground auxiliary. The years of dust exposure were based on the time they worked in the dust exposure sites. According to the production capacity, coal mines are divided into three categories: large mines (more than 1.2 million tons a year), medium mines (0.3–1.2 million tons a year) and small mines (below 0.3 million tons a year).

### Blood pressure examination

Both systolic and diastolic blood pressures were measured by trained professional nurse using mercury sphygmomanometer. The participants should have a rest for at least 10 min in a seated position before measurements. When measuring, the right arm is out-stretched at heart level using an appropriately sized cuff. The method is consistent with the National Institute for Health Care Excellence and European Society of Hypertension guidelines [12]. If readings showed abnormally high or low blood pressure, the repeated measurement was performed to evaluate consistency. One blood pressure value per participant was recorded. Standards of blood pressure were defined using the WHO/ISH recommendations: systolic measure ≥140 mmHg or diastolic measure ≥90 mmHg. In addition, participants were considered to be hypertensive if they have a diagnosis of hypertension by a physician.

### Electrocardiogram measurement

Electrocardiogram was measured with routine 12-leads electrocardiogram by electrocardiograph. The physicians were required to have professional training and the subjects were measured in supine position with cleaned skin. The results were evaluated independently by two different physicians based on the AHA/ACCF/HRS recommendations for the standardization and interpretation of the electrocardiogram (AHA/ACCF/HRS 2009).

### Pulmonary function tests

The spirometer was calibrated before tests. Participants’ age, sex, weight and height were input to the instrument. This examination was performed by a trained technician. Forced vital capacity (FVC), forced expiratory volume in the first second (FEV_1_), and FEV_1_/FVC were recorded. At least three values of all parameters were obtained for each participant, and the best value was used to assess pulmonary function. Predictive values were calculated for FEV_1_, FVC, and FEV_1_/FVC based on their respective age, sex, height, and race. The spirometry results were expressed and interpreted with percentages of predicted values (% predicted) using the American Thoracic Society and European Respiratory Society recommendations (2005) [13]. The abnormal pulmonary function group included participants with FVC < 80%, FEV_1_ < 80% or FEV_1_/FVC < 80%.

### Chest radiograph examination

All participants’ posterior-anterior chest radiograph examinations were evaluated independently by two different physicians based on China National Diagnostic Criteria for Pneumoconiosis (GBZ 70–2015), which is identical to the 1980 International Labor Organization (ILO) pneumoconiosis assessment standards.

### Statistical analysis

The continuous variables were described as mean (SD). Frequencies (n) and proportions (%) were calculated for the categorical variables. Logistic regression models were used to explore the potential risk factors for abnormal indexes, with abnormal radiograph, PF, BP, and ECG as dependent variables and sex, age, years of dust exposure, smoking, drinking, working types, and size of mine as independent variables. The statistic parameters including the odds ratios (ORs) with respective 95% confidence intervals (CIs) were calculated. The variable with *P* value < 0.05 in univariate analysis was entered the logistic regression analysis. *P* value < 0.05 was defined as statistical significance. The analysis was performed using SPSS 20.0 (SPSS Inc., Chicago, IL, USA).

## Results

### Population characteristics

The 11,061 coal miners in 2015 and 12,597 coal miners in 2016 were recruited from Jiangsu, China. In 2015, the participants included 10,032 male (90.7%) and 1029 female (9.3%); the average age of coal miners was 42.9 years (SD = 9.6) and the mean of duration of dust exposure was 15.1 years (SD = 10.4). In 2016, the participants included 11,279 male (89.5%) and 1318 female (10.5%); the average age was 42.8 years (SD = 9.2) and the mean of dust exposure time was 15.4 years (SD = 11.4). The miners with age of 41–50 years and duration of dust exposure less than 10 years accounted for the largest proportions. Most miners have the habit of smoking or drinking. The majority of miners are engaged in ground auxiliary work and employed in large coal mines (Table 1).

**Table 1:** Distribution characteristics of coal miners in 2015 and 2016

Variable	2015 *n*(%)	2016 *n*(%)
Sex		
Female	1029 (9.3)	1318 (10.5)
Male	10,032 (90.7)	11,279 (89.5)
Age, years		
≤ 30	1428 (12.9)	1512 (12.0)
31–40	2462 (22.3)	2794 (22.2)
41–50	4068 (36.8)	5031 (39.9)
> 50	3103 (28.0)	3260 (25.9)
Years of dust exposure		
≤ 10	4709 (42.6)	5640 (44.8)
11–20	2452 (22.2)	2222 (17.6)
> 20	3900 (35.2)	4735 (37.6)
Smoking		
No	5431 (49.1)	6034 (47.9)
Yes	5630 (50.9)	6563 (52.1)
Drinking		
No	3953 (35.7)	3423 (27.2)
Yes	7108 (64.3)	9174 (72.8)
Working types		
Ground auxiliary	5630 (50.9)	6762 (53.7)
Tunneling	1757 (15.9)	1514 (12.0)
Coal extraction	919 (8.3)	795 (6.3)
Pulverized coal	1520 (13.7)	1585 (12.6)
Underground auxiliary	1235 (11.2)	1941 (15.4)
Size of mine		
Large	7368 (66.6)	7680 (61.0)
Medium	2443 (22.1)	2307 (18.3)
Small	1250 (11.3)	2610 (20.7)

### Prevalence of abnormal radiograph, PF, BP and ECG in coal miners in 2015

In 2015, aberrant BP had the highest reported proportion (29.1%) in coal miners, followed by abnormal ECG (22.4%), PF (10.1%) and radiograph (2.2%). As shown in Table 2, the highest proportions of aberrant radiograph, PF, BP and ECG were all observed in coal miners with age of more than 50 years and dust exposure more than 20 years. The coal extraction workers were reported to have more radiograph problems. The proportion of abnormal PF in tunneling work was higher than other working types. Furthermore, the highest proportion of aberrant BP was found in coal miners with underground auxiliary work, and more aberrant ECG were observed in coal miners with pulverized coal work. The proportions of abnormal radiograph and PF were relatively higher among coal miners employed in the large or medium coal mines, and the proportions of abnormal BP and ECG were higher in the small coal mines.

**Table 2:** Prevalence of abnormal radiograph, PF, BP and ECG in coal miners in 2015

Variable	Radiograph *n*(%)	PF *n*(%)	BP *n*(%)	ECG *n*(%)
Sex				
Female	12 (1.2)	95 (9.2)	128 (12.4)	179 (17.4)
Male	228 (2.3)	1026 (10.2)	3091 (30.8)	2297 (22.9)
Age, years				
≤ 30	17 (1.2)	136 (9.5)	191 (13.4)	295 (20.7)
31–40	18 (0.7)	256 (10.4)	545 (22.1)	494 (20.1)
41–50	92 (2.3)	357 (8.8)	1243 (30.6)	879 (21.6)
> 50	113 (3.6)	372 (12.0)	1240 (40.0)	808 (26.0)
Years of dust exposure				
≤ 10	63 (1.3)	396 (8.4)	1009 (21.4)	957 (20.3)
11–20	17 (0.7)	158 (6.4)	749 (30.5)	477 (19.5)
> 20	160 (4.1)	567 (14.5)	1461 (37.5)	1042 (26.7)
Smoking				
No	108 (2.0)	533 (9.8)	1614 (29.7)	1221 (22.5)
Yes	132 (2.3)	588 (10.4)	1605 (28.5)	1255 (22.3)
Drinking				
No	86 (2.2)	392 (9.9)	1183 (29.9)	886 (22.4)
Yes	154 (2.2)	729 (10.3)	2036 (28.6)	1590 (22.4)
Working types				
Ground auxiliary	102 (1.8)	382 (6.8)	1611 (28.6)	1022 (18.2)
Tunneling	28 (1.6)	276 (15.7)	514 (29.3)	472 (26.9)
Coal extraction	33 (3.6)	135 (14.7)	268 (29.2)	183 (19.9)
Pulverized coal	42 (2.8)	150 (9.9)	438 (28.8)	492 (32.4)
Underground auxiliary	35 (2.8)	178 (14.4)	388 (31.4)	307 (24.9)
Size of mine				
Large	165 (2.2)	770 (10.5)	2079 (28.2)	1576 (21.4)
Medium	57 (2.3)	247 (10.1)	728 (29.8)	556 (22.8)
Small	18 (1.4)	104 (8.3)	412 (33.0)	344 (27.5)

*PF* Pulmonary function, *BP* Blood pressure, *ECG* Electrocardiogram

### Prevalence of abnormal radiograph, PF, BP and ECG in coal miners in 2016

In 2016, the prevalence of aberrant BP was also the highest (15.9%), followed by abnormal ECG (15.3%), PF (5.2%) and radiograph (4.0%). As shown in Table 3, the highest proportions of aberrant PF and ECG were observed in coal miners with age of more than 50 years and dust exposure more than 20 years. The proportions of aberrant radiograph were increased in coal miners at 41–50 years of age and more than 20 years of dust exposure, and the highest proportions of aberrant BP were identified in coal miners at 31–40 years of age and 11–20 years of dust exposure. The proportions of abnormal radiograph, PF and ECG were relatively higher among coal miners with a habit of smoking or drinking. The types of work that recorded the most radiograph and ECG problems were pulverized coal work. The proportion of abnormal PF in coal miners employed in coal extraction work was higher than those engaged in other working types. Furthermore, the highest proportion of aberrant BP was found in coal miners with underground auxiliary work. In addition, the proportions of abnormal radiograph, PF and ECG were relatively higher among coal miners employed in the large coal mines, while the proportion of abnormal BP was higher in the small coal mines.

**Table 3:** Prevalence of abnormal radiograph, PF, BP and ECG in coal miners in 2016

Variable	Radiograph *n*(%)	PF *n*(%)	BP *n*(%)	ECG *n*(%)
Sex				
Female	42 (3.2)	61 (4.6)	199 (15.1)	191 (14.5)
Male	465 (4.1)	594 (5.3)	1806 (16.0)	1732 (15.4)
Age, years				
≤ 30	9 (0.6)	25 (1.7)	178 (11.8)	110 (7.3)
31–40	127 (4.5)	84 (3.0)	579 (20.7)	413 (14.8)
41–50	232 (4.6)	311 (6.2)	815 (16.2)	841 (16.7)
> 50	139 (4.3)	235 (7.2)	433 (13.3)	559 (17.1)
Years of dust exposure				
≤ 10	158 (2.8)	132 (2.3)	778 (13.8)	801 (14.2)
11–20	101 (4.5)	147 (6.6)	556 (25.0)	329 (14.8)
> 20	248 (5.2)	376 (7.9)	671 (14.2)	793 (16.7)
Smoking				
No	206 (3.4)	206 (3.4)	1177 (19.5)	816 (13.5)
Yes	301 (4.6)	449 (6.8)	828 (12.6)	1107 (16.9)
Drinking				
No	62 (1.8)	114 (3.3)	438 (12.8)	391 (11.4)
Yes	445 (4.9)	541 (5.9)	1567 (17.1)	1532 (16.7)
Working types				
Ground auxiliary	207 (3.1)	323 (4.8)	758 (11.2)	867 (12.8)
Tunneling	31 (2.0)	39 (2.6)	310 (20.5)	209 (13.8)
Coal extraction	51 (6.4)	85 (10.7)	175 (22.0)	128 (16.1)
Pulverized coal	117 (7.4)	71 (4.5)	283 (17.9)	416 (26.2)
Underground auxiliary	101 (5.2)	137 (7.1)	479 (24.7)	303 (15.6)
Size of mine				
Large	331 (4.3)	423 (5.5)	911 (11.9)	1200 (15.6)
Medium	90 (3.9)	117 (5.1)	493 (21.4)	326 (14.1)
Small	86 (3.3)	115 (4.4)	601 (23.0)	397 (15.2)

*PF* Pulmonary function, *BP* Blood pressure, *ECG* Electrocardiogram

### Potential risk factors associated with abnormal radiograph, PF, BP and ECG of coal miners in 2016

The logistic regression models were then used to explore the risk factors of abnormal radiograph, PF, BP and ECG (Table 4). It is found that age, years of dust exposure and working types were the potential risk factors of abnormal radiograph and BP. The risk factors for aberrant PF included years of dust exposure, smoking and working types. In addition, the coal miners employed in the small mines had higher risk of PF and BP compared with miners in the large mines. Meanwhile, the observed risk factors of aberrant ECG were age, drinking and working types.

**Table 4:** Logistic regression analysis for potential risk factors associated with abnormal indexes of coal miners in 2016

Variable	Radiograph	PF	BP	ECG
	OR(95% CI)	OR(95% CI)	OR(95% CI)	OR(95% CI)
Sex				
Female	1.00	1.00	1.00	1.00
Male	1.28 (0.92–1.78)	1.06 (0.80–1.41)	1.01 (0.86–1.19)	1.00 (0.85–1.18)
Age, years				
≤ 30	1.00	1.00	1.00	1.00
31–40	4.05 (1.92–8.54)^*^	0.88 (0.52–1.49)	1.19 (0.94–1.51)	1.92 (1.48–2.50)^*^
41–50	2.71 (1.24–5.90)^*^	1.30 (0.75–2.25)	1.49 (1.14–1.94)^*^	2.13 (1.60–2.84)^*^
> 50	1.75 (0.75–4.09)	1.16 (0.62–2.18)	1.44 (1.02–2.03)^*^	1.98 (1.39–2.81)^*^
Years of dust exposure				
≤ 10	1.00	1.00	1.00	1.00
11–20	1.26 (0.97–1.64)	2.93 (2.27–3.79)^*^	1.82 (1.60–2.08)^*^	0.89 (0.77–1.03)
> 20	1.70 (1.33–2.17)^*^	2.64 (2.09–3.34)^*^	1.29 (1.12–1.48)^*^	0.98 (0.86–1.11)
Smoking				
No	1.00	1.00	1.00	1.00
Yes	1.20 (0.83–1.72)	1.92 (1.34–2.71)^*^	0.93 (0.61–1.50)	1.16 (0.95–1.42)
Drinking				
No	1.00	1.00	1.00	1.00
Yes	2.20 (1.61–3.00)^*^	1.26 (0.97–1.64)	1.11 (0.95–1.31)	1.20 (1.03–1.40)^*^
Working types				
Ground auxiliary	1.00	1.00	1.00	1.00
Tunneling	0.97 (0.65–1.47)	0.91 (0.57–1.43)	2.05 (1.77–2.38)^*^	1.01 (0.85–1.19)
Coal extraction	2.16 (1.57–2.97)^*^	2.43 (1.88–3.14)^*^	2.15 (1.78–2.59)^*^	1.27 (1.04–1.56)^*^
Pulverized coal	2.56 (2.02–3.24)^*^	0.95 (0.73–1.23)	1.75 (1.51–2.04)^*^	2.37 (2.07–2.70)^*^
Underground auxiliary	1.80 (1.41–2.30)^*^	1.50 (1.21–1.85)^*^	2.67 (2.35–3.05)^*^	1.23 (1.07–1.42)^*^
Size of mine				
Large	1.00	1.00	1.00	1.00
Medium	0.95 (0.70–1.28)	1.75 (1.34–2.28)^*^	1.88 (1.61–2.20)^*^	1.09 (0.93–1.29)
Small	0.90 (0.61–1.17)	1.88 (1.39–2.55)^*^	1.95 (1.66–2.29)^*^	1.14 (0.96–1.36)

*PF* Pulmonary function, *BP* Blood pressure, *ECG* Electrocardiogram

^*^P < 0.05

### Description of diagnosed CWP of coal miners in 2015 and 2016

A total of 80 subjects diagnosed with CWP in 2015–2016, which occupied 0.34% of the coal miners. In 2015, the average age at diagnose was 63.6 years (SD = 11.4) and the mean of duration of dust exposure in new diagnosed CWP was 25.6 years (SD = 7.9). In 2016, the average diagnostic age was 57.7 years (SD = 11.5) and the mean of dust exposure time in new diagnosed CWP was 17.7 years (SD = 8.6).

## Discussion

To date, few reports have mentioned the health surveillance data of Chinese coal miners. Based on this, we analyzed the occupational examination data of coal miners collected from 2015 to 2016 in Jiangsu, China. In this study, the highest proportions of aberrant PF and radiograph were found among older coal miners. The variation in working types among mining sectors and duration of dust exposure are likely to contribute to the different health risks, and a better understanding of these differences could help us to prevent CWP. Our work also found that the greater proportions of abnormal radiograph and PF were observed among miners with long-term underground tenure. In addition, the proportion of abnormal PF was associated with the size of mines, which may due to the different conditions of protective measures and production capacity [14]. Despite the evident need for more health data specific to each mining sector and duration of dust exposure, there appear to be elevated risks of pulmonary disease across mining in general [15]. The results further supported that the potential coal miners with pulmonary function impairment, who have not developed into CWP, may need special attention.

When evaluating coal miners’ health, it is important to consider other health concerns, and not simply focus on pulmonary disease. In this study, aberrant BP had the highest reported proportion. Hypertension, which occurs almost simultaneous with overweight/obesity and dyslipidaemia, is intensively related to cardiovascular disease [16]. Hypertension has been reported as the main health risk for dust exposure workers. Xu et al. clearly indicated that the welders under welding fumes exposure have higher blood pressure than the controls [17]. The obesity and hypertension were the known risk factors for cardiovascular disease. Based on the Enhanced Coal Workers’ Health Surveillance Program, Casey et al. pointed out that the prevalence of obesity and hypertension in US coal miners was higher than the US adult population [11], which indicated a high need for cardiovascular health assessment and intervention in coal miners. In our work, the highest proportion of aberrant BP (20.7%) was identified in coal miners at 31–40 years of age in 2016, which was higher than the average values about 17.9% of men and 8.8% of women at 35–39 years of age in China [18]. The high prevalence of hypertension may cause the increase of dyslipidaemia and diabetes in coal miners. Lewington et al. indicated that the prevalence of hypertension was correlated with age [18], which was consistent with our study. Additionally, we observed that underground front-line miners with long-term dust exposure had the highest proportion of aberrant BP than ground workers. Maybe it is because the underground miners have higher labor intensity. Fan et al. indicated that the proportions of obesity and overweight in coal miners were 17.6 and 39.0%, respectively; these are all higher than the Chinese national average values [19]. It is undoubted that the overweight and obesity were risk factors for hypertension. Therefore, hypertension in Chinese coal miners also need to be concerned.

Epidemiological studies found that dust particles were the likely mining-related risk factor for the morbidity and mortality of cardiovascular disease [20]. Exposure to dust particles causes aberrant heart rate and heart rate variability, which are parameters for the heart autonomous control. Moreover, exacerbations of cardiac function damage were observed within individuals with diabetes and hypertension after particles exposures [21]. It also has been noted that exposure to high concentration of dust particles in coal mine for a long time might trigger myocardial infarctions [22]. In this study, we observed that the highest proportion of aberrant ECG was found in coal miners with age of more than 50 years. These results demonstrated that coal workers with long time dust exposure are generally at higher risk for abnormal ECG. The most consistent evidence with this result was that the abnormal ECG rate of ground auxiliary was lower than in coal miners with tunneling, coal extraction, pulverized coal and underground auxiliary work. It can be concluded that the different types of work affected the cardiac health of the miners. Although the lack of reference data limits our ability to determine whether abnormal ECG rates in our sample differs from the general population, these results indicated that long exposure to coal dust may be associated with changes in cardiac function in miners. Furthermore, analysis of this sample is an initial step in assessing the cardiac health of coal miners. Future research is needed to further systematically examine the cardiac health of miners based on more clinical indexes and to confirm the correlation between the coal dust exposures and cardiac health. Results from this study could help identify and focus future health and safety initiatives, as well as engineering controls targeted at decreasing dust exposure concentration, with the ultimate goal of improving the health of coal miners.

Our study had several limitations. First, the data were collected based on the Occupational Disease Monitoring and Occupational Health Risk Assessment Program, so for most miners, blood pressure was recorded at one time. Although the measurement was repeated when it showed abnormal, these repeated measurements were not performed on all subjects. This method have been used in some convincing surveys, including the Enhanced Coal Workers’ Health Surveillance Program among US coal miners [11] and a research about occupational exposure to particles and blood pressure from southern Sweden [17]. In the National Health and Nutrition Examination Surveys, Handler et al. demonstrated that when the initial blood pressure value is normal, upward reclassification from a non-hypertensive to pre-hypertensive or Stage 1 hypertensive is relatively uncommon [23]. We recognized that the measurement of blood pressure for more than one point may obtain more exact results. This issue will be noticed in our future study. Second, spirometric and ECG abnormalities were not specific indexes for CWP, which may limit the usefulness of the findings for understanding occupational aetiology of CWP. However, they will add information for future researches to evaluate the health hazards of coal miners.

## Conclusion

The abnormal radiograph, PF, BP and ECG are general and highlighted health problems of coal miners in China. Demographic characteristics (advanced age, smoking and drinking), working characteristics (types of work, years of dust exposure and size of mines) were closely related to health status of coal miners. In addition to the regular physical examination, feasible health promotion and protective facilities should be adopted to guarantee coal miners’ health.

## Additional file

Additional file 1: The Occupational Health Questionnaire. This questionnaire surveys demographic, working characteristics and the data of occupational examinations. (DOCX 24 kb)

## Data Availability

The datasets used during the current study are available from the corresponding author on reasonable request.

## Abbreviations

BP: Blood pressure
CWP: Coal workers’ pneumoconiosis
ECG: Electrocardiogram
PF: Pulmonary function
