# Supplementary material for: Effects of occupational exposure to dust on chest radiograph, pulmonary function, blood pressure and electrocardiogram among coal miners in an eastern province, China
Source: BMC Public Health. 2019 Sep 5;19:1229. doi: 10.1186/s12889-019-7568-5 (PMC6728990; doi:10.1186/s12889-019-7568-5)
Supplement: Supplementary file 1 — The Occupational Health Questionnaire. This questionnaire surveys demographic, working characteristics and the data of occupational examinations. (DOCX 24 kb) [file 12889_2019_7568_MOESM1_ESM.docx]

| Date | Radiograph Number | Electrocardiogram Number |
| --- | --- | --- |
|  |  |  |

Category of Harmful Factors：

Examination Category: Before Employ □

Employ □

Retired □

Occupational Health Questionnaire

Employing Unit：

Unit Code：□□□□□□□□□□

Unit Size：

Name： Gender：

ID Number：

Work Number：

Signature：

Date：

**1. General Item**

Place of Birth：

Age：

Nation：

Marriage：

Education：

Phone：

Initial Date of Employ：

Total Years of Employ：

Years of Dust Exposure：

Types and Names of Dust：

**2. Occupational History**

| Date | Employing Unit | Workshop | Working Type | Protective Facilities |
| --- | --- | --- | --- | --- |
|  |  |  |  |  |
|  |  |  |  |  |
|  |  |  |  |  |

**3. Medical History**

**4. Family Medical History**（History of hereditary, hemorrhagic, malignant tumor and other diseases）

**5. History of Occupational Disease**

Type：

Degree of Occupational Disease：

Date of Diagnosis：

Department of Diagnosis：

Whether or not to heal：

**6. Gestational History**

|  | Pregnant Time | Gender | Natural Birth | Preterm Birth | Abortion |
| --- | --- | --- | --- | --- | --- |
| First Child |  |  |  |  |  |
| Second Child |  |  |  |  |  |
| Third Child |  |  |  |  |  |

**7. Personal History**

Smoking：No smoking□

Occasionally smoking□

Often smoking cigarettes/day, Total years

Drinking：No drinking□

Occasionally drinking□

Often drinking ml/day, Total years

**8. Others**

| **9. Physical Sign** | | | | | | | | | | | | | | | | | |  |  |
| --- | --- | --- | --- | --- | --- | --- | --- | --- | --- | --- | --- | --- | --- | --- | --- | --- | --- | --- | --- |
| Item | | | | | | | | Result | | | Item | | | | Result | | |  |  |
| General Condition | | Height | | | | | |  | | | Surgery | | Thyroid | |  | | |  |  |
|  |  | Weight | | | | | |  | | |  |  | Lymph Node | |  | | |  |  |
|  |  | Blood pressure | | | | | | mmHg | | |  |  | Spine | |  | | |  |  |
|  |  | Sphygmus | | | | | |  | | |  |  | Four Limb | |  | | |  |  |
|  |  |  | | | | | |  | | |  |  | Breast | |  | | |  |  |
| Otolaryngology | | Outer ear | | | | | |  | | |  |  | Prostate | |  | | |  |  |
|  |  | Hearing | | | Left | | |  | | |  |  |  | |  | | |  |  |
|  |  |  |  |  | Right | | |  | | |  |  |  | |  | | |  |  |
|  |  | Tympanic membrane | | | | | |  | | | Dermatology | | Skin | |  | | |  |  |
|  |  | Nose | | | | | |  | | |  |  | Fingernail | |  | | |  |  |
|  |  | Throat | | | | | |  | | |  |  | Hair | |  | | |  |  |
|  |  | Tonsil | | | | | |  | | |  |  |  | |  | | |  |  |
|  |  |  | | | | | |  | | | Internal Medicine | | Heart | |  | | |  |  |
|  |  |  | | | | | |  | | |  |  | Lung | |  | | |  |  |
| Ophthalmology | | Vision | | | | Left | |  | | |  |  | Liver | |  | | |  |  |
|  |  |  |  |  |  | Right | |  | | |  |  | Spleen | |  | | |  |  |
|  |  | Conjunctiva | | | | | |  | | |  |  |  | |  | | |  |  |
|  |  | Cornea | | | | | |  | | |  |  |  | |  | | |  |  |
|  |  | Pupil | | | | | |  | | |  |  |  | |  | | |  |  |
|  | |  | | | | | |  | | |  | |  | |  | | |  |  |
| **10. Laboratory Examination** | | | | | | | | | | | | | | | | | |  |  |
| Item | | | | | | | | | | Result | Item | | | | | | Result |  |  |
| Blood | | Leukocyte ×10^9^/L | | | | | | | |  | Basophil % | | | | | |  |  |  |
|  |  | Neutrophil % | | | | | | | |  | Erythrocyte ×10^12^/L | | | | | |  |  |  |
|  |  | Lymphocyte % | | | | | | | |  | Hemoglobin g/L | | | | | |  |  |  |
|  |  | Eosnophils % | | | | | | | |  | Thrombocyte ×10^9^/L | | | | | |  |  |  |
|  |  | Monocyte % | | | | | | | |  |  | | | | | |  |  |  |
| Urine | | Urine Protein | | | | | | | |  | Liver Function | | | Alanine Aminotransferase | | |  |  |  |
|  |  | Urine Sugar | | | | | | | |  |  |  |  | Total Bilirubin | | |  |  |  |
|  |  | Erythrocyte | | | | | | | |  |  |  |  | Total Protein | | |  |  |  |
|  |  | Leukocyte | | | | | | | |  |  |  |  | White/Globulin | | |  |  |  |
|  |  |  | | | | | | | |  |  |  |  | HBsAg | | |  |  |  |
|  |  |  | | | | | | | |  |  |  |  | Anti-HBs | | |  |  |  |
| Renal Function | | | | | Creatinine | | | | |  |  |  |  | HBeAg | | |  |  |  |
|  |  |  |  |  | Urea Nitrogen | | | | |  |  |  |  | Anti-HBe | | |  |  |  |
|  | | | | | | | | | |  |  |  |  | Anti-HBc | | |  |  |  |
| **11. Functional and Special Examination** | | | | | | | | | | | | | | | | | | | |
| Item | | | | | | | | Result | | | | | Item | | Result | | | | |
| Chest Radiograph | | | | No： | | | |  | | | | | Electrocardiogram | |  | | | | |
| Pulmonary Function | | | | FVC % | | | |  | | | | | B-mode Ultrasound  (Liver, Gallbladder, Spleen, Kidney) | |  | | | | |
|  |  |  |  | FEV_1_ % | | | |  | | | | |  |  |  |  |  |  |  |
|  |  |  |  | FEV_1_/FVC % | | | |  | | | | |  |  |  |  |  |  |  |
|  |  |  |  |  | | | |  | | | | |  |  |  |  |  |  |  |
|  |  |  |  |  | | | |  | | | | |  |  |  |  |  |  |  |
|  | | | |  | | | |  | | | | |  |  |  |  |  |  |  |
| **12. Conclusions** | | | | | | | | | | | | | | | | | | | |
| Results and Comments | | | | | | | Doctor’s signature:  Date: | | | | | | | | | | | | |
| Examination Institution | | | | | | | Date: | | | | | | | | | | | | |
